# Design of CMOS-compatible metal–insulator–metal metasurfaces via extended equivalent-circuit analysis

**DOI:** 10.1038/s41598-020-74849-5

**Published:** 2020-10-21

**Authors:** Alexander Dorodnyy, Stefan M. Koepfli, Alexander Lochbaum, Juerg Leuthold

**Affiliations:** Institute of Electromagnetic Fields, ETH Zurich, Zurich, Switzerland

**Keywords:** Integrated optics, Mid-infrared photonics, Optical sensors, Optoelectronic devices and components

## Abstract

Photonic metasurfaces compatible with large-scale production such as CMOS are of importance because they promise cointegration of electronics with photonics for detection, communication and sensing. The main challenges on the way of designing such metasurfaces are: (1) large variety of possible geometrical shapes of metasurface elements that makes finding the most appropriate shape difficult; (2) poor compatibility of available electronic layer stacks with photonics. In this paper we show how to address both of these challenges utilizing extended equivalent-circuit analysis. In a first step we classify the behavior of different metasurfaces using the equivalent circuit. We discover that metasurfaces that use inverted-dipole resonator type exhibit higher tolerance to dielectric spacer thickness, higher angular stability and have similar resonance quality-factor as other types. In the second step we utilize the equivalent-circuit scheme to efficiently optimize the parameters of inverted-dipole based metasurfaces for a layer stack such as given in a CMOS process. Finally, as an example we demonstrate how an inverted-cross structure can be adapted to a commercial 110 nm CMOS process with Al metal layers. We measured peak absorption above 90% at center wavelength around 4 µm with quality factor of approximately 5 and angular stability larger than 60°.

## Introduction

Metal-dielectric metasurfaces find common use in optical sensing and communication applications since they allow to shape optical spectra, wave-fronts and polarizations^1–6^. They can also be used to increase the light-matter interaction for strong absorption/emission^7–10^ or non-linear effects^4,11–13^, to create beam splitters^14,15^, selective reflectors^16,17^, holograms^18–20^, polarizers^21–23^ and for various other applications such as chiral photonic structures and chemical sensors^24–26^. Metal–insulator–metal (MIM) metasurfaces are of particular importance since they can be monolithically integrated with electronics in the same layer stack^27–29^ and benefit from readily available high-yield large-scale production technologies, such as Complementary-Metal–Oxide–Semiconductor (CMOS), enabling low-cost optoelectronic devices^30,31^. MIM metasurfaces in particular are well suited for creating perfect absorption on selected wavelength(s)^7–9^. However, the challenge remains to incorporate a perfect absorber with the desired absorption characteristics into a given layer stack.

To solve the inverse problem of creating a MIM metasurface with given absorption characteristics, it is often more efficient to use the equivalent-circuit analysis rather than to use a full-wave solver for a 3D-model. The equivalent circuit allows to decrease the simulation time and apply a number of analytical derivations^32–37^. This analysis is possible because metasurface elements are typically deeply sub-wavelength in size. Hence, the electromagnetic field within the metasurface can be roughly considered a plane-wave, and the propagation of the light within the metasurface can be approximated by a propagation of a single mode via an effective medium. The latter then can be shown to be analogous to the propagation of an electrical signal through a transmission line^32^. In the transmission line model, the electric field corresponds to the voltage in the transmission line and the magnetic field to the current. Resonant metal layers correspond to inductive, capacitive and resistive elements, and dielectric spacers to transmission line segments (see Supplementary Information S1).

Generally, in order to use the equivalent-circuit model, one needs to find a relation between the geometrical structure parameters and their equivalent-circuit representations. Then, it is possible to translate a 3D-model into an equivalent circuit, find the optimal parameters of this circuit such that they fit into desired performance margins and, in a last step, to translate it back to 3D-structure parameters. This however, requires sufficiently accurate analytic model to translate a set of equivalent circuit parameters to a structure geometry and back. Such analytic models are often not available. A more flexible solution is to analyze the behavior of an equivalent circuit in general (without restricting it to parameters yielded by specific resonator geometries) by classifying all possible outcomes and ranges of parameter values that yield these results. Once done such analysis allows to determine how close to the best performance each particular geometry is and which types of geometries would give better results.

In this work we demonstrate an extended equivalent circuit analysis that allows the exploration of large metasurface design spaces without extensive full-wave simulations. The model provides intuitive insights into the behavior of various resonator types. Also, it allows to predict key characteristics of a metamaterial absorber such as maximal achievable absorption, maximal achievable Q-factor and reachable angular stability. Our equivalent circuit model enables fast and efficient optimization of the resonator design and an estimation of the metasurface characteristics. Finally, we present a characterization results of a MIM metasurface realized in CMOS technology and designed with the help of extended equivalent circuit analysis. The metasurface were optimized for the highest Q factor, absorption and angular stability achievable within the given constraints.

### Absorption diagram of metal–insulator–metal perfect-absorber

**Figure 1:**
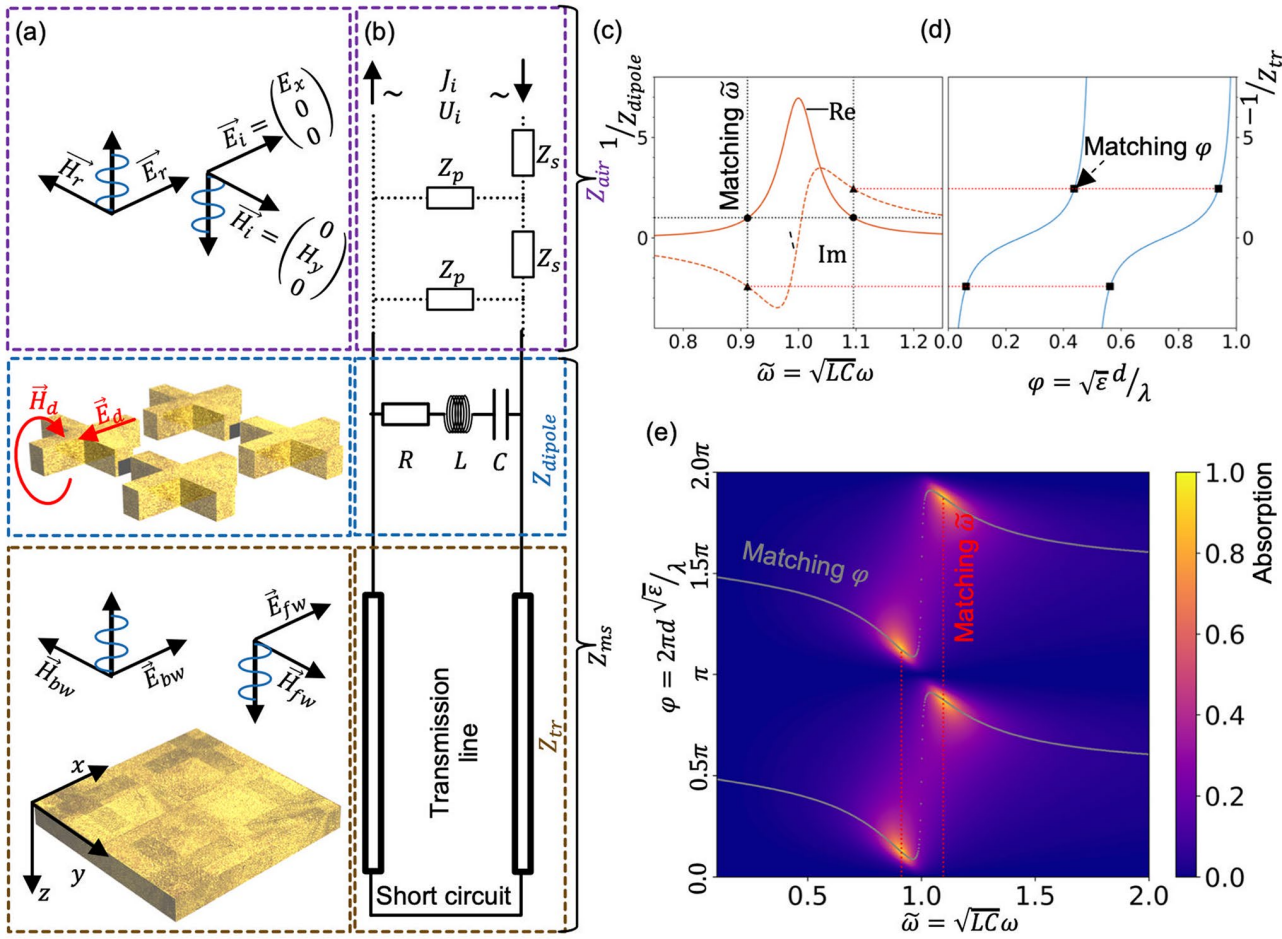
Equivalent scheme for MIM-type MPA with dipole-type resonators in shape of crosses. **(a)** Explosion view of the MPA and the corresponding electric and magnetic fields that are created under an incident plane-wave excitation. The schematic is divided in three sections: (1) free-space from where the incident wave (${\overrightarrow{E}}_{i}, {\overrightarrow{H}}_{i}$) impinges onto the resonator; (2) the front-side resonators; (3) an intermediate dielectric terminated with a conductive plane. At the resonator layer, the charge oscillation induced fields (${\overrightarrow{E}}_{d}, {\overrightarrow{H}}_{d}$) are indicated. Behind the resonator two plane-waves are indicated: one traveling towards the backplane (${\overrightarrow{E}}_{fw}, {\overrightarrow{H}}_{fw}$) and one in the opposite direction (${\overrightarrow{E}}_{bw}, {\overrightarrow{H}}_{bw}$). **(b)** Equivalent circuit corresponding to the metasurface shown in **(a)**. Parts corresponding to the same sections are highlted with the same colors as in **(a)**. **(c)** Real and imaginary parts of dipole-type resonator impedance (inverted values). Points where real part equals to 1 are shown by circles, corresponding imaginary parts are shown by triangles. **(d)** Imaginary part of transmission-line impedance (inverted value with minus sign). Dashed lines extending from subplot **(c)** to subplot **(d)** show values of the imaginary part for which resulting normalized impedance of the metasurface has a real value equal to 1, i.e. $\mathrm{Re}\left({\stackrel{\sim }{Z}}_{dipole}\right)=1$. Corresponding points at subplot **(d)** are marked by squares. **(e)** Absorption diagram showing the absorption dependence on the normalized frequency $\stackrel{\sim }{\omega }$ ($C,L$ are capacitance and inductance corresponding to the resonator layer) and the accumulated phase $\varphi$ ($\varepsilon ,d,\lambda$ are dielectric permittivity, dielectric thickness and light wavelength correspondingly). Matching lines show values of $\stackrel{\sim }{\omega }$ and $\varphi$ that correspond to the matching points marked at subplots **(c,d)**, at their intersection the perfect absorption is acquired. See Supplementary Information (S2) for parameters of the equivalent circuit.

Several types of MIM metasurfaces have been demonstrated to be used as perfect absorbers tunable to specific wavelengths^7–9,38^. Figure 1 shows an example of a metamaterial perfect absorber (MPA) based on a cross-shape resonator structure. The MIM stack consists of a reflective metal backplane, a transparent dielectric spacer-layer and the metallic resonator-layer. In this article we will consider resonant layers that consist of resonant periodic unit-cells. The top metal layer provides the resonant response to an external electromagnetic excitation. The underlying insulation layer and the backplane provide impedance correction for the perfect matching with the excitation wave impedance. Due to the underlaying metal backplane MPAs of that type do not transmit any light and instead either reflect or absorb it. The absorption $\mathcal{A}$ commonly has one or few peaks at certain frequency(es) where $\mathcal{A}\approx 1$ which are the points of perfect absorption (further on we refer to them as matching points because this is where the input impedance of the metasurface matches that of the external media). Depending on the resonator layer the matching points might exist only for few specific spacer thicknesses between the resonator layer and the backplane. We call the dependence of the absorption on frequency and phase-shift (accumulated by a plane-wave on the way between the resonator and the backplane) the absorption diagram (see Fig. 1e for an example). The phase-shift is calculated per single trip between the resonant layer and the backplane and is further denoted as $\varphi$ and referred to as the accumulated phase. The absorption diagram is the key to understand metasurface MPA behavior and, as it is shown further, can be reconstructed by the usage of the equivalent-circuit method to get a better understanding of the MPAs physics.

Equivalent-circuit method is a powerful tool to understand and be able to predict the resonant behaviour of MIM-metasurface absorbers. The method takes advantage of the analogy between a plane-wave propagation in an isotropic media and a propagation of a signal through a transmission line (see Supplementary Information (S1) for the details). Figure 1a,b shows how a correspondence can be built between an actual structure of MIM-metasurface and its equivalent-circuit model. In case of the cross-type resonator (as shown in Fig. 1), the resonance part can be represented by a capacitor $C$ that mimics the charge accumulation from a cross bar acting as a dipole antenna that is parallel to the incident wave electric field. Charge oscillations in the dipole have an inertia due to self-inductance and non-zero mass of the carriers. This inertia can be expressed in the equivalent-circuit by an addition of the inductive element $L$ that would prevent instant current changes. Additionally, we need to consider that a dipole has losses that need to be added as a corresponding resistive element $R$. The fields that are created by the charge oscillations correspond, in this case, to the following: the electric field ${E}_{d}$ of the dipole corresponds to the voltage on the capacitor, the magnetic field ${H}_{d}$ of the dipole corresponds to the current that flows through the capacitor. Outside of the resonator layer we assume that field propagates in four plane-waves: $({E}_{i},{H}_{i})$ is the incoming excitation, $({E}_{r},{H}_{r})$ is the reflected wave, $({E}_{fw},{H}_{fw})$ is the wave propagating forward from resonator to the backplane and $({E}_{bw},{H}_{bw})$ is the one propagating in the opposite direction. These four waves are corresponding to the signals propagating through adjacent pats of the equivalent circuit.

To find the point at which the reflection reaches zero ($\mathcal{R}=0$), we require that the Fresnel equation for the reflection meets the condition

1 $$\mathcal{R}={\left|\frac{{Z}_{air}-{Z}_{ms}}{{Z}_{air}+{Z}_{ms}}\right|}^{2}=0.$$

This holds if and only if ${Z}_{air}={Z}_{ms}$, where ${Z}_{air}\approx 377$ Ohm is the air impedance and ${Z}_{ms}$ is the impedance of the metasurface (see Fig. 1b). In other words, the impedances of air and metasurface need to match.

The metasurfaces impedance ${Z}_{ms}$ needed for solving Eq. (1) is illustrated in the equivalent-circuit drawn in Fig. 1b. ${Z}_{ms}$ consists of two parallelly connected parts: ${Z}_{dipole}$ corresponding to the resonant layer and ${Z}_{tr}$ corresponding to the dielectric spacer and backplane.

2 $$\frac{1}{{Z}_{ms}}=\frac{1}{{Z}_{dipole}}+\frac{1}{{Z}_{tr}}$$

The resonator impedance according to the scheme shown in Fig. 1b is

3 $${Z}_{dipole}=i\left(\frac{1}{C\omega }-L\omega \right)+R.$$

Here we used the fact that impedances of a capacitor and an inductor can be expressed as $i/\omega C$ and $-i\omega L$ correspondingly.

The dielectric spacer and the backplane can be represented by a transmission line shorted at the end. We neglect losses in the backplane since we know from full-wave simulations that they are considerably lower than losses in the resonant layer and do not give any qualitative contribution to the results.

The impedance of a transmission-line combined with short circuit can be expressed as

4 $${\mathrm{Z}}_{tr}={\mathrm{Z}}_{line}\frac{1-{e}^{2i\varphi }}{1+{e}^{2i\varphi }},$$

where $\varphi$ is the accumulated phase per a single trip from dipole to the backplane. The equation is derived from the known formula for translating an impedance ${Z}_{0}$ over the transmission line with impedance ${Z}_{line}$ ^39^

5 $${\mathrm{Z}}_{tr}={Z}_{line}\frac{({Z}_{line}+{Z}_{0})+{e}^{2\alpha L}({Z}_{0}-{Z}_{line})}{({Z}_{line}+{Z}_{0})-{e}^{2\alpha L}({Z}_{0}-{Z}_{line})},$$

where $L$ is the length of the line and $\alpha$ is a propagation constant. In our case ${Z}_{0}=0$ because the transmission line ends with the short circuit. We substituted here $\alpha L$ with $i\varphi$ assuming the transmission line is lossless and considering the propagation constant to be strictly imaginary.

Subsequently, we use the normalized frequency $\stackrel{\sim }{\omega }=\omega \sqrt{LC}$ in order to get a simplified expression

6 $${Z}_{dipole}=i\sqrt{\frac{L}{C}}\left(\frac{1}{\stackrel{\sim }{\omega }}-\stackrel{\sim }{\omega }\right)+R.$$

In the normalized units the dipole impedance depends on two parameters ${\sqrt{L/C}}$ and $R$ instead of three ($L,C,R$). To further simplify the analysis, we can normalize all the impendence values with respect to the air impedance ${Z}_{air}$

7 $${\stackrel{\sim }{Z}}_{dipole}=i{\stackrel{\sim }{Z}}_{LC}\left(\frac{1}{\stackrel{\sim }{\omega }}-\stackrel{\sim }{\omega }\right)+\stackrel{\sim }{R},$$

where $${\stackrel{\sim }{Z}}_{dipole}={Z}_{dipole}/ {Z}_{air}$$, $${\stackrel{\sim }{Z}}_{LC}={\sqrt{L}/{C}/ {Z}_{air}}$$ and $$\stackrel{\sim }{R}=R/{Z}_{air}.$$

We perform the same normalization for the transmission line impedance

8 $${\stackrel{\sim }{Z}}_{tr}={\stackrel{\sim }{Z}}_{line}\frac{1-{e}^{2i\varphi }}{1+{e}^{2i\varphi }}.$$

Furthermore, we will later assume that ${\stackrel{\sim }{Z}}_{line}=1$. This assumption is based on the fact that the right part of Eq. (8) does not change its behavior qualitatively, subsequently giving no qualitative difference in the results in the reasonable window of ${\stackrel{\sim }{Z}}_{line}$, as it is shown in the Supplementary Information (S5).

One can obtain from the numerator of Eq. (1) the condition for zero-reflection which in case of the introduced normalized impedances is

9 $$\frac{1}{{\stackrel{\sim }{Z}}_{dipole}}+\frac{1}{{\stackrel{\sim }{Z}}_{tr}}=1.$$

One can notice from Eq. (8) that the value of ${\stackrel{\sim }{Z}}_{tr}$ is strictly imaginary, so that Eq. (9) can be written as two separate equations

10 $$\mathrm{Re}\left({\stackrel{\sim }{Z}}_{dipole}\right)=1$$

11 $$\mathrm{Im}\left(\frac{1}{{\stackrel{\sim }{Z}}_{tr}}\right)=-\mathrm{Im}\left(\frac{1}{{\stackrel{\sim }{Z}}_{dipole}}\right).$$

Satisfying both Eqs. (10, 11) yields the perfect matching and therefore 100% absorption.

Figure 1c–e illustrates the impedance matching between the metasurface and the air for a dipole type resonator (such as the resonator example of Fig. 1a). Figure 1c shows a plot of the real and imaginary parts of the impedance of the dipole array (value inverted), i.e. $1/{\stackrel{\sim }{Z}}_{dipole}$ as per Eq. (7) as a function of $\stackrel{\sim }{\omega }$ (with exemplary values of ${\stackrel{\sim }{Z}}_{LC}=1.91,$ $\stackrel{\sim }{R}=0.14$). Equation (10) requires that the real part equals to 1 for the perfect matching with the air. There are two solutions of Eq. (10) and they are marked by black circles. The imaginary parts of the dipole impedance are fixed due to the choice of the real part values (marked by triangles). Figure 1d is a plot of the imaginary part of ${-1/\stackrel{\sim }{Z}}_{tr}$ as a function of accumulated phase $\varphi$, see Eq. (8). Perfect impedance matching requires that the imaginary parts of both $1/{\stackrel{\sim }{Z}}_{dipole}$ and ${-1/\stackrel{\sim }{Z}}_{tr}$ are equal. The corresponding imaginary values of the transmission line can be found graphically by the dotted lines that extend from Fig. 1c to Fig. 1d (marked by squares). The coupling principle that was illustrated via the equivalent-circuit impedances can be also qualitatively interpreted in terms of electric and magnetic fields (see Supplementary Information (S1)).

The absorption can now be calculated from $\mathcal{A}=1-\mathcal{R}$ with Eq. (1). Figure 1e displays the absorption as a function of the normalized frequency $\stackrel{\sim }{\omega }$ and the accumulated phase $\varphi$ (the absorption diagram). There are two insights gained from a construction of the absorption diagram. Firstly, the sensitivity of the absorption to changes in $\varphi$ and $\stackrel{\sim }{\omega }$ has implications on the angular stability. A change of the angle of the incident light, will increase the accumulated phase $\varphi$ in proportion to the frequency $\stackrel{\sim }{\omega }$, therefore, the smaller influence change in $\varphi$ has on the resonance—the more angularly stable it is. It worth to note that there is an influence of the angle of incidence on the equivalent-circuit parameters such as capacitance^32,33^, however great correspondence between equivalent-circuit, full-wave simulations and experiments indicate that for at least angles up to around 40° it is not significant. Secondly, for a given frequency, the maximum absorption will always correspond to a value of $\varphi$ for which the Eq. (10) is satisfied. The latter can be strictly derived from Eq. (1) if one keeps in mind that change in $\varphi$ only affects the imaginary part matching (Eq. 11).

### Classification of resonator structures

**Figure 2:**
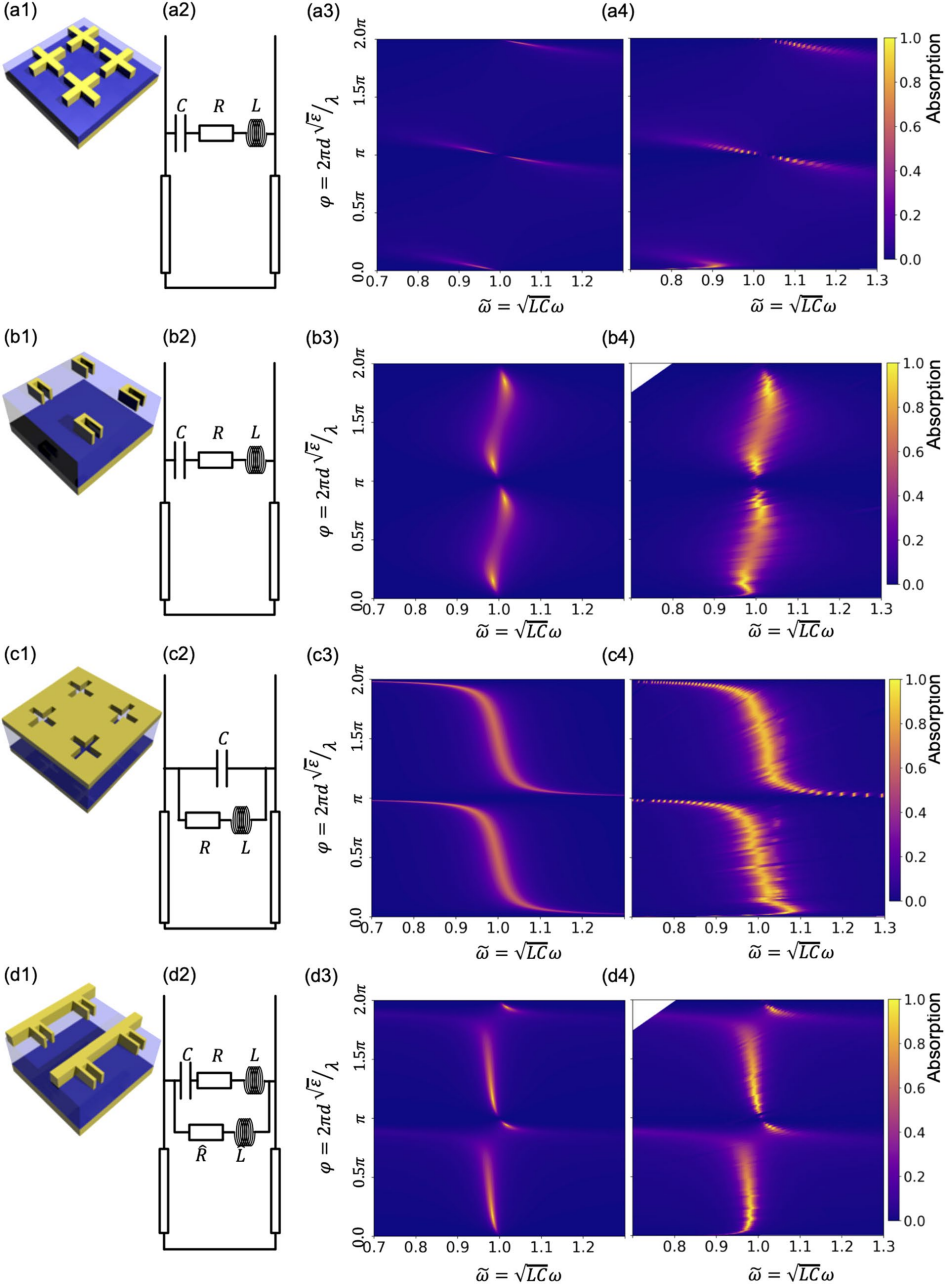
Equivalent circuit and full-wave simulation results for a set of characteristic resonator structures. Schematic representation of four unit cells of **(a1)** a cross-shape dipole **(b1)** an U-shape dipole (all results shown for electric field polarized across the slit in "U"), **(c1)** an inverted-cross dipole and **(d1)** combination of U-shape dipole with interconnection lines that function as non-localized current-path channels similar as it is for the inverted dipole (same electric field polarization is chosen as for the case of U-shape dipole). **(a2–d2)** Corresponding equivalent-circuits. **(a3–d3)** Typical absorption diagrams of each resonator type calculated with the equivalent-circuit model. **(a4–d4)** Same diagrams obtained via full-wave simulation. By observing the absorption diagram behavior of each resonator types conclusions can be drawn concerning its performance for given conditions. For example, structures **(c,d)** exhibit absorption resonance that are tolerant to the accumulated phase $\varphi$ shift. The latter indicates that they have better angular stability of absorption. See Supplementary Information (S2) for parameters of the structures and their equivalent circuits.

Let us now extend the absorption diagram analysis to other than dipole resonator types. Figure 2 presents a summary of two basic resonator types—the dipole type and the inverted-dipole type shown in Fig. 2a1,b1 and c1 respectively. We chose to show two structures of the same dipole type, namely cross and U-shape dipoles to demonstrate how absorption diagrams can vary depending on the structure of choice within the same type of resonators. For the chosen parameters (see Supplementary Information (S2) for details). The U-shape dipole has a larger inductance and a higher loss leading to different positions of the absorption maxima on the $\varphi$-axis and broader absorption peaks. One can notice however that although positions and widths of the absorption maxima changed from the U-shape to the cross structures the topology of the absorption diagram is the same. It has two absorption maxima around a node point at $\varphi =\pi , \stackrel{\sim }{\omega }=1.0$ which repeats periodically on the $\varphi$-axis with a period of $\pi$. For the inverted-dipole structure we have a qualitatively different dependence. A single absorption maximum is stretched along a line that has a nearly vertical segment at $\stackrel{\sim }{\omega }=1.0$. This nearly vertical segment indicates a presence of absorption resonance that will occur on the same frequency for a large range of possible dielectric thicknesses and for the same reason it indicates an angular stability. Tolerance towards thickness variations and angular stability of the inverted-dipole absorption diagram are important for creating CMOS-compatible MIM absorber and will be used in the following.

It is worth to note how we compose the inverted-dipole equivalent-circuit. To understand it we need to start with a homogeneous metal layer. We then introduce a gap that is equivalent to a capacitor where the capacitance is provided by charge accumulations on the gap sides. The induced current that flows around the gap is not localized like in case of the dipole but instead flows through the entire metal layer and all gaps included in it. The equivalent circuit element for such a resonator can therefore be represented as shown in Fig. 2c2. In parallel to the introduced capacitance the current can flow around the gap and only a fraction of all carried charge would accumulate on the gap sides.

The equivalent circuit impedance belonging to the inverted dipole then is

12 $${\stackrel{\sim }{Z}}_{inv\_dipole}=\frac{\frac{i{\stackrel{\sim }{Z}}_{LC}}{\stackrel{\sim }{\omega }}\left(\stackrel{\sim }{R}-i{\stackrel{\sim }{Z}}_{LC}\stackrel{\sim }{\omega }\right)}{\stackrel{\sim }{R}-i{\stackrel{\sim }{Z}}_{LC}\stackrel{\sim }{\omega }+\frac{i{\stackrel{\sim }{Z}}_{LC}}{\stackrel{\sim }{\omega }}},$$

where the same normalization is made as for the case of the direct dipole.

Having now equivalent-circuits for both the dipole and the inverted dipole structures we can also compose a combination of them as shown in Fig. 2d. The structure shown in Fig. 2d1 is a combination of U-shape dipoles with an interconnection line that connects the U-shapes in the direction of the external electric field. Due to this interconnection we obtain both types of oscillation modes in the same structure: Primarily localized as in the case of the dipole and distributed as in the case of the inverted dipole. The resulting equivalent circuit, see Fig. 2d2, is a combination of dipole and inverted-dipole equivalent circuits. The circuit includes the same serially connected capacitance, inductance and resistance as for the dipole that are placed in parallel with inductance and resistance of the interconnecting line in the same manner as in the inverted dipole equivalent-circuit. The absorption diagram obtained with the combined structure reproduces well the one obtained with full-wave simulations, see Fig. 2d3,d4, which confirms its validity. One can also notice that this absorption diagram combines features of dipole and inverted-dipole absorption diagrams. Same as the dipoles it has pairs of absorption maxima situated at around $\varphi =\pi N, \stackrel{\sim }{\omega }=1.0, N\in {\mathbb{N}}$ node points. But in this case the maxima are distorted and repeat the shape of inverted-dipole absorption maximum. To illustrate the behavior of dipole, inverted-dipole and combination modes, Supplementary Information (S6) shows electric filed, magnetic field and currents for each of the discussed resonator types.

We can formulate more general rules for describing more complicated resonator structures as follows. Charge oscillations in an arbitrary periodic structure under plane-wave excitation would be either localized within each unit cell or would have currents flowing through all of them. Elements with localized charge oscillations correspond to sequentially placed capacitances and inductances (like a dipole), whereas elements with oscillations connected by parallel lines/channels correspond to parallel arrangement (like the inverted dipole).

Overall the full-wave simulations are in very good agreement to the equivalent-circuit calculations. The model only shows larger deviations to the full-wave solutions if the accumulated phase is low ($\varphi <0.1\pi )$. The threshold for the applicability of the equivalent circuit model thus is reached at around $\varphi =0.1\pi$. Below this threshold the spectrum of the metasurface is influenced considerably by the near filed coupling between metal layers, and the equivalent-circuit needs to be further adopted to describe its behavior properly.

### Performance mapping in equivalent-circuit parametric-space

In this section we analyze the behavior of the inverted-dipole structure within CMOS constraints to answer two questions: (1) How good can it perform in terms of absorption maxima and resonance quality factors, (2) for a particular design that we would use, how close is it to the potential best performance within the given CMOS constraints.

**Figure 3:**
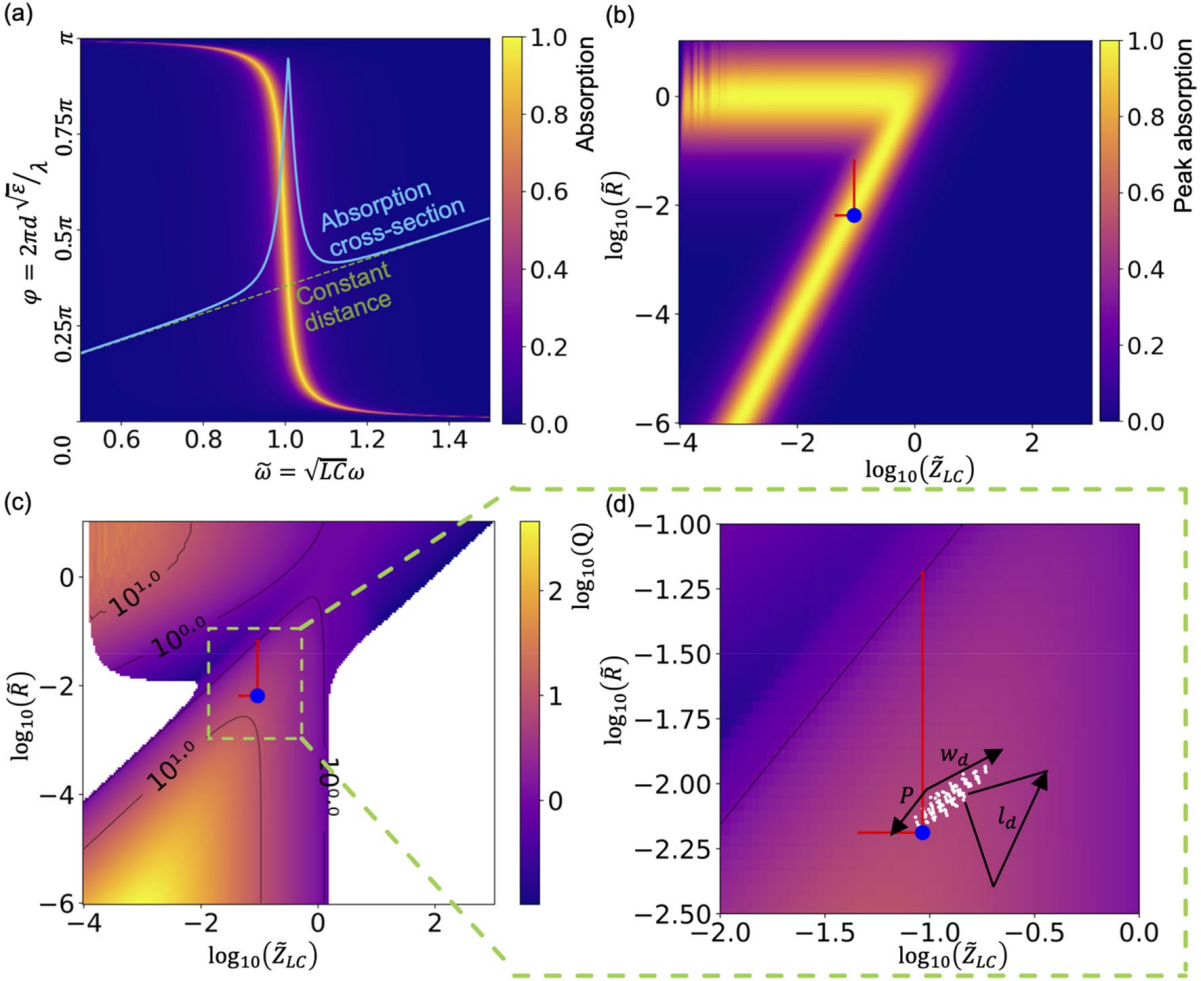
Absorption-diagram parameter plots of a MIM metasurface with the inverted-dipole resonator structure with fixed dielectric thickness corresponding to 500 nm SiO_2_ as found in the chosen CMOS process. **(a)** Typical absorption diagram for an inverted-dipole resonator for the 500 nm SiO_2_ dielectric spacer. The dashed green line shows corresponding dependence of $\varphi$ on $\stackrel{\sim }{\omega }$. Solid blue line drawn above the green line shows the dependence of the absorption on $\stackrel{\sim }{\omega }$. **(b)** Dependence of the peak absorption on the parameters of the equivalent circuit ${\stackrel{\sim }{Z}}_{LC}$ and $\stackrel{\sim }{R}$. **(c)** Dependence of Q-factor of the absorption resonance on ${\stackrel{\sim }{Z}}_{LC}$ and $\stackrel{\sim }{R}$. Blue dots on subplots **(b,c)** show best parameters of the equivalent-circuit that were fitted for an inverted-dipole structure within the CMOS layer-stack. Vertical red lines indicate ranges in which resistance of the resonator would change by a factor of 10, horizontal red lines indicated a range in which capacitance would change by factor 2. The ranges were chosen based on an expectation that adhesion layer losses are higher than estimated based on database permittivity values, and that due to the SiO_2_ environment capacitance of resonators can be increased by around a factor 2. **(d)** Results of 140 simulations (white dots) that are aimed to determine which parameter range can inverted-cross structure cover in the given CMOS constraints (${w}_{d},{l}_{d},P$ were varied in ranges of [200, 400], [1100, 1400], [1250, 1700] nm correspondingly). $P$ corresponds to the periodicity of the structure, ${w}_{d}$ to the slit width and ${l}_{d}$ to the slit length.

For the fabrication we used 110 nm feature-size Al/SiO_2_/Al CMOS process with fixed 500 nm SiO_2_ spacing between the layers and fixed Al-layer thickness of around 250 nm. Resonators and the backplane were implemented in M4 and M3 layers correspondingly. Within these constraints we attempt to find the optimum MIM resonator layer. For this sake, one can notice that the absorption of a simple metasurface (such as a dipole or an inverted-dipole resonator type) depends on four parameters: ${\stackrel{\sim }{Z}}_{LC},\stackrel{\sim }{R},\stackrel{\sim }{\omega },\varphi$. To each pair of values of ${\stackrel{\sim }{Z}}_{LC}$ and $\stackrel{\sim }{R}$ belongs an absorption diagram (where the absorption is a function of $\stackrel{\sim }{\omega }$ and $\varphi$). The absorption diagram then may be analyzed to issue key parameters (such as maximal reachable absorption, width of the absorption resonance etc.) that can be achieved for the chosen ${\stackrel{\sim }{Z}}_{LC}$ and $\stackrel{\sim }{R}$. The analysis of absorption peak and Q-factor for MIM MPA with inverted-dipole resonator type is shown in Fig. 3. For each pair of ${\stackrel{\sim }{Z}}_{LC}$ and $\stackrel{\sim }{R}$ one could build an absorption diagram as shown in Fig. 3a. From such diagram one can find the maximal absorption point values of ${\stackrel{\sim }{\omega }}_{max}$ and ${\varphi }_{max}$, that corresponds to given dielectric thickness and for these conditions extract the achievable absorption peaks and Q-factor. If one then scans across a range of possible values of ${\stackrel{\sim }{Z}}_{LC}$ and $\stackrel{\sim }{R}$ one receives color-plots that give an overview of achievable values for key characteristics of a metasurface.

Figure 3b,c show absorption-diagram parameter graphs plotted for a MIM metasurface with the inverted-dipole type resonator. We started by picking values of ${\stackrel{\sim }{Z}}_{LC}$ and $\stackrel{\sim }{R}$ within some reasonable ranges of (${10}^{-3},{10}^{3}$) and (${10}^{-6},{10}^{1}$). For each pair of values, we constructed an absorption diagram such as shown in Fig. 3a. On these diagrams we first find the point of maximal absorption for given SiO_2_ thickness of 500 nm (marked as (${\stackrel{\sim }{\omega }}_{max},{\varphi }_{max}$) in Fig. 3a). For this sake we assumed SiO_2_ permittivity to be constant and equal to 2 (rough average value of silica permittivity across different databases and deposition techniques for wavelengths around 4 μm). A dashed green line in Fig. 3a shows an accumulated phase dependence on the normalized frequency for a case of the given dielectric thickness between the resonator and the backplane. The blue line in Fig. 3a shows the corresponding absorption dependence on the frequency. From this dependence the peak absorption and the Q-factor can be extracted. Subsequently, the procedure is repeated for each pair of ${\stackrel{\sim }{Z}}_{LC}$ and $\stackrel{\sim }{R}$ within the range. This then provides the color-plots for peak absorption and Q-factor plots depicted in Fig. 3b,c.

Once this equivalent-circuit analysis is done we cross-check it with 3D-model simulations. This step is necessary as not all values of equivalent circuit parameters ${\stackrel{\sim }{Z}}_{LC}$ and $\stackrel{\sim }{R}$ are physically implementable for the given CMOS constraints. We performed 140 evaluations with the full-wave simulator where we varied three resonator parameters (periodicity, cross length and cross width) and extracted the equivalent circuit parameters ${\stackrel{\sim }{Z}}_{LC}$ and $\stackrel{\sim }{R}$ for a resonant metal layer with surrounding adhesion layers (see Supplementary Information (S3) for details of the fitting procedure). The extracted parameters are shown in Fig. 3d, where we show a zoom in on a region of the Q-factor plot shown in Fig. 3c and added the points for the 140 evaluations of the resonator. We can now easily select the best pair of parameters and, furthermore, see how each parameter allows us to move in the ${\stackrel{\sim }{Z}}_{LC}$-$\stackrel{\sim }{R}$ space. One can notice from Fig. 3c that in order to increase the Q-factor one would need to either decrease feature sizes (${l}_{d}$, ${w}_{d}$) or to increase the period of the structure ($P$). The length of the gap ${l}_{d}$ is limited from below by around a quarter of the wavelength of interest and the width of the gap ${w}_{d}$ by the feature-size of CMOS. Period $P$ cannot be much larger than the wavelength of the resonance as it affects the coupling between the resonant layer and the surrounding media leading to increasing reflection. Blue dots in Fig. 3b,c represent parameters ${\stackrel{\sim }{Z}}_{LC}$ and $\stackrel{\sim }{R}$ corresponding to the best possible structure within the given CMOS constraints. Additional vertical and horizontal red lines stretching from the best set of fitted parameters (blue dots in Fig. 3b,c) show ranges of an expected change in the equivalent-circuit parameters due to unaccounted losses in the stack (mostly related to adhesion layers and interlayer surface quality, which although were included in the model can vary in properties between the real layers and the database data) and capacitance increase of the resonator due to neighboring dielectric layers that were not included in the full-wave simulation of only the resonator layer. The resistance window was chosen somewhat arbitrary and is corresponding to exactly one order of magnitude for illustration purposes. The capacitance window is chosen to correspond to change of dielectric environment up to 2 × in permittivity (corresponding to 2 × larger capacitance) which is a reasonable guess for SiO_2_ dielectric layers.

Focusing on the feasible performance range for CMOS structures within the chosen process (see the blue dot in Fig. 3b,c) we conclude that we should be able to produce a metamaterial absorber with an absorption peak value above 90%, Q factor in the range of 10 and high angular stability. We can further conclude that there is no hidden-local maxima of absorption or Q-factor outside of the one that we find our structure to fall into. That is an indication that we are not likely to obtain a better resonator unless we are able to reduce losses by an order of magnitude. The losses are strongly connected with the adhesion layers present in the CMOS process and we cannot lower them considerably within the given layer-stack. We therefore conclude that our structure should be close to the optimal design for the given CMOS process.

Worth to note that, although the presented analysis focuses on absorptive MIM metasurfaces, it can be applied in similar fashion to other metal-dielectric stacks with periodic or isotropic layers that satisfy two conditions: (1) weak near-field coupling between the metal layers; (2) does not involve propagation of more than a single mode in each of the dielectric layers. The first condition, as simulations show, is satisfied when distance between metals in units of phase-shift at the wavelength of interest is above $\sim 0.1\pi$. The second condition is satisfied when the metasurface period is below the diffraction limit. Note also, that although the multi-mode structures are out of the scope of the consideration presented in this article, it is in general possible to address multi-mode regimes with the equivalent-circuit analysis^32^.

### Characterization of CMOS-produced perfect-absorber

**Figure 4:**
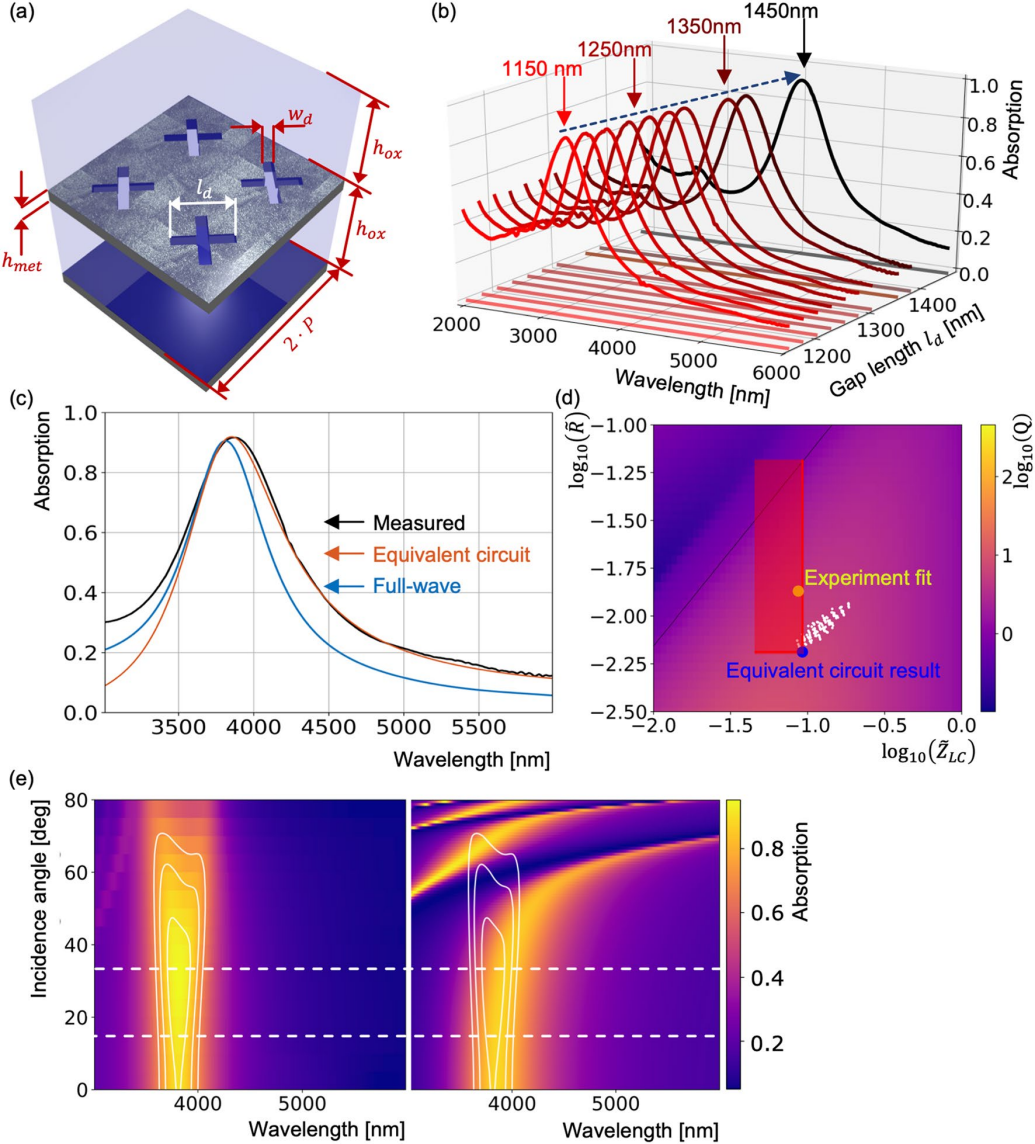
Comparison between experimental measurements, full-wave simulations and the equivalent-circuit prediction. **(a)** Schematics of the inverted-dipole resonator type MIM metasurface that were produced in M3–M4 layers of a CMOS process. The 500 nm SiO_2_ layer is preserved above the top metal layer to account for mechanical strain. **(b)** Experimental absorption spectra for various lengthes ${l}_{d}$ as marked at top. **(c)** Comparison of the measurement (black line) for the average cross length (1250 nm) with simulation for the same dimensions (blue line) and equivalent circuit with best fitting capacitane and resistance values taken from within the range that was originally considered, as highlighted in subplot **(d)**. **(d)** Subplot analogous to Fig. 3d with added point of the best match between the experiment and the equivalent-circuit (yellow dot). **(e)** Absorption spectra simulated with full-wave solver for an average cross length (1250 nm) and different angles of incidence from 0 to 80° with a step of 5° (left) and the same spectra calculated with the equivalent circuit (right). One can notice that the equivalent circuit results overlap well with full-wave simulations up until around 40°.

To verify that we could indeed produce the MIM metasurface with characteristics predicted by the equivalent-circuit analysis the designed structure was produced with the 110 nm CMOS-process. The fabricated metasurfaces were then characterized in a MID-IR FTIR setup. A Cassegrain objective was used to illuminate and collect light between 25 and 35° of incidence (see Supplementary Information (S4) for more details). Figure 4 shows schematics and simulated/measured spectra of the inverted cross resonator metasurface produced in CMOS.

Figure 4a shows a schematic of the fabricated inverse dipole MIM-metamaterial. We show four unit-cells and indicate all relevant parameters (${h}_{met}=250$, ${w}_{d}=200$, ${h}_{ox}=500$ and $P=1400$ nm). We swept the length of the slits ${l}_{d}$ from 1150 to 1450 nm, which tunes the absorption peak position as shown in measurement results Fig. 4b. We use the slit length ${l}_{d}=1250 \mathrm{nm}$ to verify that we indeed were able to realize the simulated spectrum. Figure 4c shows the measured spectrum, the full-wave spectrum and the spectrum calculated with the equivalent circuit. A quality factor in the range of 10 with an absorption peak above 80% was predicted from the equivalent-circuit analysis (according to the range highlighted in Fig. 4d). The full 3D-model predicted a quality factor of 5.5 and an absorption peak at 90%. Ultimately, in the fabricated structure a quality factor of 4.0 and an absorption peak of 90% were measured. Since fitting the parameters of the equivalent-circuit leaves a range of uncertainty for the parameters as highlighted in Fig. 4d, we also show a corrected spectra where the parameters were chosen (from within the marked range) such that they fit experiment the best. Losses that are represented by resistance in the equivalent circuit were increased by a factor 2 compared to the original estimation and the capacitance value was increased by 7%. One can see that although the equivalent-circuit only gives an estimation on the range of possible outcomes the obtained results fit well within that range. The deviation of the equivalent-circuit and measured results at the lower wavelengths is due to additional diffraction modes that are excited inside the MIM stack at lower wavelengths that are not taken into account by the equivalent circuit (which only considers the lowest order mode with no diffraction). These measurements demonstrate that the designed structures can be produced in CMOS to yield tunable high-absorption metasurfaces. The quality factor of the resonance is moderate but, as the analysis shows, it is close to the highest one achievable with the chosen CMOS stack (illustrated in Fig. 4d). In addition to the spectral predictions, we also investigate the expected angular stability of the stack both with full-wave simulations and the equivalent circuit method. Figure 4e shows the high angular stability of the layer-stack (left), however, the equivalent circuit results (right) are only comparable to around 40°. The deviation occurs due to the parameters of the resonant mode such, as the capacity^32,33^, which has a dependence on the excitation angle. Large deviations from the original angle for which the fitting was done start to have an influence on the accuracy of the calculations. However, covering a larger angular range is possible by refining the estimation at certain angles where the deviation becomes too large. For our setup the validity range of the equivalent circuit overlaps well with the measurement setup angular range.

## Conclusions

In this article we showed how extending the standard equivalent-circuit analysis by a parametric analysis of absorption diagrams, we are able to find the most suitable type of resonator for MIM MPA that yields close to the best possible performance within given processing constraints. In particular, we demonstrated that inverted-dipole type resonator-structures yield the best angular stability for the largest range of possible dielectric thicknesses. The inverted-dipole resonator is therefore determined to be the most suitable to be implemented within limitations of the chosen 110 nm feature-size CMOS-process. For the implementation we chose inverted-cross structures. We then showed that by mapping the MPA performance characteristics, such as maximum absorption and Q-factor of the resonance, against normalized equivalent circuit parameters that the performance that we reach with the particular chosen geometry is close to the best possible within the given process constraints.

Worth to note that, although in this article we focused on the optimization of the absorption for finding best metasurface type for the chosen CMOS-process, it is possible to build parametric maps for any quantities that can be extracted from an absorption diagram. For example, one could optimize a selective metasurface that reflects light on one frequency and transmits on another creating a dichroic filter.

By performing FTIR reflection measurements on the fabricated metasurface we demonstrate that following the theoretical predictions based on the equivalent-circuit analysis we obtained no-postprocessing (apart from removing few CMOS layers) CMOS-produced metasurface with over 90% absorption, quality factor around 5 and angularly stable to at least 35°. Great correspondence between theory and experiment further indicates that the absorption should be stable up to at least 60°.

We believe that suggested analysis method can be a powerful tool for analyzing MIM MPA behaviors to find best possible solutions for given design constraints by severely cutting down computational cost. It also gives intuitive insights into the various metasurface behaviors that help to choose the most optimal type of structure for a given application.

The usage of the equivalent-circuit allows for considerable acceleration of the analysis, as for example, the parametric map for the inverted-cross type resonator takes only 10 min to calculate with the equivalent circuit approach, while a single full-wave simulation of one absorption spectrum takes around 2 min. Therefore, an absorption diagram with the same resolution requires several hours (~ 5 h for 150 dielectric thickness points and ~ 1000 h for a full parametric map). The computational cost is therefore severely cut and the methods enables the fast generation of additional insights into this interesting artificial material class.

## Methods

FTIR setup included: broadband source, KBr beam splitter, infrared microscope with Cassegrain objective (angular range between 25 and 35°) and MCT detector. For every spectrum 32 measurements with 4 cm^-1^ resolution were performed and averaged for the final result. To get the absolute values of the absorptivity golden-mirror reference was used with assumption of that it reflects 100% of the incoming light.

For full-wave simulations CST Microwave Studio FEM frequency-domain solver was used. The equivalent-circuit code was implemented in Python.

Simulations were performed on standalone servers: with Intel Xeon Platinum 8160 processor running CentOS 7 for the equivalent-circuit; and with Intel Xeon E5-2637 v4 processor running Windows 10 for the full-wave analysis. In both cases RAM capacity exceeded by far the requirements of the simulations.

## Supplementary information

Supplementary Information.
