# Supplementary material for: Design of CMOS-compatible metal–insulator–metal metasurfaces via extended equivalent-circuit analysis
Source: Sci Rep. 2020 Oct 21;10:17941. doi: 10.1038/s41598-020-74849-5 (PMC7578050; doi:10.1038/s41598-020-74849-5)
Supplement: Supplementary file 1 — Supplementary Information. [file 41598_2020_74849_MOESM1_ESM.docx]

Design of CMOS-Compatible Metal-Insulator-Metal Metasurfaces via Extended Equivalent-Circuit Analysis

**Supplementary Information**

Alexander Dorodnyy^1^, Stefan M. Koepfli^1^, Alexander Lochbaum^1^ and Juerg Leuthold^1^

^1^Institute of Electromagnetic Fields, ETH Zurich, 8092 Zurich, Switzerland

## S1. Equivalence Between the Plane-Wave and the Equivalent-Circuit Signal Propagations

To see the analogy between Maxwell's equations and the equations describing propagation of a signal through a transmission line we can substitute $\left[ E,H,\varepsilon,\mu\right]$ with $\left[ V,J,\frac{ic}{\omega Z_{p}},\frac{iZ_{s}c}{\omega} \right]$ for an equivalent set of equations. Here $\left[ E,H,\varepsilon,\mu\right]$ represent electric field, magnetic field, permittivity and permeability, and $\left[ V,J,Z_{p},Z_{s},\omega\right]$ correspond to the voltage in the line, current in the line, parallel impedance per unit length, serial impedance per unit length and frequency of the signal (see Fig. S1(b) for the illustration of serial and parallel impedances $Z_{s}$ and $Z_{p}$). We start with Maxwell's equations written for a plane-wave with electric field along the x-axis and magnetic field along the y-axis

| $\left\{ \begin{aligned} \vec{\nabla}\times\vec{E}=-\frac{\mu}{c}\frac{\partial\vec{H}}{\partial t} \\ \vec{\nabla}\times\vec{H}=\frac{\varepsilon}{c}\frac{\partial\vec{E}}{\partial t} \end{aligned} \right.$ | (S1.1) |
| --- | --- |
| $\left\{ \begin{aligned} \frac{\partial E_{x}}{\partial z}=-\frac{\mu}{c}\frac{\partial H_{y}}{\partial t} \\ \frac{\partial H_{y}}{\partial z}=-\frac{\varepsilon}{c}\frac{\partial E_{x}}{\partial t}. \end{aligned} \right.$ | (S1.2) |

Equation (S1.2) can be obtained from (S1.1) by confining ourselves to non-zero field components $E_{x}$ and $H_{y}$.

Ohm's law for a transmission line as depicted in the free space propagation layer Fig. S2(b), one write

| $\left\{ \begin{aligned} \frac{\partial V}{\partial z}=-Z_{s}J \\ \frac{\partial J}{\partial z}=-\frac{V}{Z_{p}} \end{aligned} \right.$ | (S1.3) |
| --- | --- |
| $\left\{ \begin{aligned} \frac{\partial V}{\partial z}=-\frac{iZ_{s}}{\omega}\frac{\partial J}{\partial t} \\ \frac{\partial J}{\partial z}=-\frac{i}{\omega Z_{p}}\frac{\partial V}{\partial t}, \end{aligned} \right.$ | (S1.4) |

where the current in the line would drop over the parallel elements and the voltage over the serial elements. Equation (S1.4) can be obtained from (S1.3) if we assume harmonic oscillation of both current and voltage at frequency $\omega$, such that $J\left( t \right),V\left( t \right)\sim e^{-i\omega t}$.

As one can see equations describing plan-wave propagation (S1.2) are analogous to those for a transmission line (S1.4). We highlighted analogous elements in equations (S1.2, S1.4) with the same colors.

Making use of the same analogy between the plane-wave propagation and the transmission line we now can introduce a real geometry and substitute it with its equivalent-circuit elements. Each of the dielectric media would be substituted by a transmission line whereas metal layers of sub-wavelength thicknesses would be substituted by lumped elements.





Fig. S1 Components of the metasurface and corresponding equivalent circuit elements. (a) Structure of MIM metasurface with cross-type resonators and electromagnetic fields inside the structure. (b) Corresponding equivalent-circuit elements.

To see how the coupling mechanism between the free-space mode and the metasurface can be described in terms of electric and magnetic fields one can consider the boundary condition at the resonant layer interface. For that purpose, we can put down the sum of fields before and behind the “Resonator layer”. In the absence of reflected fields we have

| $\left\{ \begin{matrix} \vec{E}_{i}+\vec{E}_{d}=\vec{E}_{d}+\vec{E}_{fw}+\vec{E}_{bw} \\ \vec{H}_{i}+\vec{H}_{d}=-\vec{H}_{d}+\vec{H}_{fw}+\vec{H}_{bw} \end{matrix} \right..$ | (S1.5) |
| --- | --- |

Here $\vec{E}_{i}$ and $\vec{H}_{i}$ are incident electric and magnetic fields, $\vec{E}_{d}$ and $\vec{H}_{d}$ electric and magnetic fields of the metasurface resonant layer created by the dipoles. $\vec{E}_{fw}$,$\vec{H}_{fw}$,$\vec{E}_{bw}$,$\vec{H}_{bw}$ are electric and magnetic fields of the waves propagating to and from the backplane within the metasurface, as illustrated in Fig. S1(a). The electric field of the dipole is symmetric and cancels out of equations (S1.5), whereas the magnetic field of the dipole has opposite signs and does not cancel out. We can therefore regroup equations (S1.5) as follows

| $Z_{air}=\frac{E_{i}}{H_{i}}=\frac{E_{fw}+E_{bw}}{H_{fw}+H_{bw}-2H_{d}}=Z_{ms},$ | (S1.6) |
| --- | --- |

where $Z_{air}$ denotes the impedance of the incident wave in the air. The right side of equation (S1.6) is the input impedance of the metasurface that we denote as $Z_{ms}$, see Fig. S1(b). The condition of zero reflection can than simply be written as $Z_{ms}=Z_{air}$. The metasurface impedance comprises of two contributions. On the one hand, the impedance of the dipole $Z_{dipol}$ and the impedance of the dielectric spacer with backplane $Z_{tr}$ that are connected in parallel

| $\frac{1}{Z_{ms}}=\frac{1}{Z_{dipol}}+\frac{1}{Z_{tr}}.$ | (S1.7) |
| --- | --- |

The impedance of the dielectric spacer and the backplane is

| $Z_{tr}=\frac{E_{fw}+E_{bw}}{H_{fw}+H_{bw}}.$ | (S1.8) |
| --- | --- |

By comparing equations (S1.6-S1.8) we find that the impedance of the resonant layer should be

| $Z_{dipole}=-\frac{E_{fw}+E_{bw}}{2H_{d}}=\frac{E_{i}}{-2H_{d}},$ | (S1.9) |
| --- | --- |

where we used the fact that at the boundary $E_{i}=E_{fw}+E_{bw}$ from equations (S1.5). The impedance of the resonator layer is equal to the ratio of external filed incident on the layer to the difference of the magnetic fields on two sides of it. This is equivalent to a parallelly connected lumped-element impedance, which is equal to the ratio between the applied voltage and the current difference on two sides of the element.

## S2. Structure and Equivalent-Circuit Parameters





Fig. S2 Schematics of U-shape, inverted cross and interconnected U-shape resonators with in-plane geometrical parameters (excluding the period) marked. For case of cross dipoles the parameter notation is chosen in the same way as for the inverted cross (A - width of the cross-bar, B - length of the cross-bar).

Here we collect all parameters of the structure geometry and equivalent-circuits that are presented in the main article. Fig. S2 shows the notation that we chose for the in-plane set of geometry parameters.

Table 1. Parameters of the structures simulated with the equivalent circuit and with full-wave solver.

| Figure and Structure | Parameter Name | Value |
| --- | --- | --- |
| Fig. 1(c,d,e) Dipole Equivalent-Circuit | $\tilde{R}$ | $0.14$ |
|  | $\tilde{Z}_{LC}$ | $1.91$ |
| Fig. 2(a) Cross-Dipole Equivalent-Circuit | $\tilde{R}$ | $6.70\cdot{10}^{-3}$ |
|  | $\tilde{Z}_{LC}$ | $1.04$ |
| Fig. 2(a) Cross-Dipole Structure | A | 260 nm |
|  | B | 1700 nm |
|  | Metal Height | 98 nm |
|  | Period | 2000 nm |
| Fig. 2(b) Dipole Equivalent-Circuit for U-shape Dipole | $\tilde{R}$ | $0.21$ |
|  | $\tilde{Z}_{LC}$ | $1.43\cdot{10}^{1}$ |
| Fig. 2(b) U-shape Structure | A | 1050 nm |
|  | B | 250 nm |
|  | C | 80 nm |
|  | D | 250 nm |
|  | Metal Height | 150 nm |
|  | Period | 1850 nm |
| Fig. 2(c) Inverted-Dipole Equivalent-Circuit | $\tilde{R}$ | $7.53\cdot{10}^{-4}$ |
|  | $\tilde{Z}_{LC}$ | $4.14\cdot{10}^{-2}$ |
| Fig. 2(c) Inverted-Dipole Structure | A | 50 nm |
|  | B | 1423 nm |
|  | Metal Height | 52 nm |
|  | Period | 2216 nm |
| Fig. 2(d) Combined-Structure Equivalent-Circuit | $\tilde{R}$ | $6.46\cdot{10}^{-2}$ |
|  | $\tilde{Z}_{LC}$ | $7.61$ |
|  | $\tilde{\hat{R}}$ | $4.55\cdot{10}^{-3}$ |
|  | $\frac{\hat{L}}{L}$ | $6.46\cdot{10}^{-2}$ |
| Fig. 2(d) Combined-Structure | A | 957 nm |
|  | B | 260 nm |
|  | C | 697 nm |
|  | D | 260 nm |
|  | E | 200 nm |
|  | Metal Height | 98 nm |
|  | Period | 1850 nm |
| Fig. 4c Inverted-Dipole Equivalent-Circuit | $\tilde{R}$ | $1.35\cdot{10}^{-2}$ |
|  | $\tilde{Z}_{LC}$ | $8.7\cdot{10}^{-2}$ |

## S3. Resonator Parameter Fitting

Fig. S3 summarizes the resonator parameter fitting for the basic shapes such as the cross, the U-shape, the inverted-cross and the interconnected U-shapes. Subplots (a1)-(d1) show full structure of MIM metasurfaces for listed resonator types. Subplots (a2)-(d2) show corresponding equivalent circuits. Subplots (a3)-(d3) show comparison of 3D-simulation of reflection and absorption of a resonator in air (only the top layer without the backplane or the dielectric spacer) with the same dependences calculated with the equivalent circuit. Parameters of each equivalent circuit were optimized to fit the best corresponding 3D-simulations for the case of only considering the resonator layer. Note that in this case near-field coupling between the neighboring elements of the metasuface is already taken into account since the coupling is present in the simulation of the standalone resonant metal layer. Subplots (a4)-(d4) show absorption diagrams (of the full MIM-structure) obtained for the parameter sets that were optimized considering only the resonator layer. Subplots (a5)-(d5) show for comparison the same absorption diagrams but calculated with 3D-simulation. For a proper fitting both reflections and absorptions simulations need to be considered. Including both quantities allows one to derive the single optimal value for the equivalent-circuit parameters that fits well for only the resonator layer and for the full MIM structure.





Fig. S3 (a1)-(d1) Schematics of four types of resonators (cross, U-shape, inverted corss and interconnected U-shape). (a2)-(d2) Corresponding equivalent circuits. (a3)-(d3) Corresponding reflection/absorption responses for resonator layers in air (suspended in the air with no backplane or the spacer layer). Solid lines show 3D simulation results, dotted lines show fitted equivalent-circuit results. Parameters of the equivalent circuit were fitted by using a Nelder-Mead optimizaiton algorithm. (a4)-(d4) Absorption diagrams calculated with fitted parameters of the equivalent circuits. (a5)-(d5) Absorption-diagrams calculated by means of full-wave solver.

Once can notice that for sufficiently large accumulated phase values ($\varphi>0.1\pi$) equivalent circuit results (Fig. S3(a4)-(d4)) show good agreement with full-wave simulations (Fig. S3(a5)-(d5)). Therefore, once the parameters of the resonator are fitted, they can be used to recreate the absorption diagram without any additional full-wave 3D-model simulations based on the equivalent circuit only.

## S4. FTIR Measurement Setup

The setup that was used to measure the fabricated structure performance is schematically shown in Fig. S4.





Fig. S4 Schematics of the experimental FTIR setup. The angles of the Cassegrain objective are: $\theta_{1}\approx25$^o^ and $\theta_{2}\approx35$^o^.

The setup includes a broadband IR-source, an actuated mirror in a Michelson interferometer configuration, a KBr beam splitter to send the beam to the sample and also send the reflected beam to a cooled MCT detector. A Cassegrain objective (15x magnification) is used to illuminate the sample and collect the reflected light. The aperture was restricted to 200 x 200 $\mu m^{2}$. A protected gold mirror was used as a reference to normalize the spectrum of the broadband IR-source. Final spectra were recorded with a resolution of 4 cm^-1^ and each shown spectrum is an average of 32 consecutive measurements.

## S5. Permittivity of Dielectric Spacer





Fig. S5 (a) Inverted-cross absorption diagram (the same as in Fig3. (a)). (b) Absorption diagram for the same equivalent-circuit but with dielectric-spacer permittivity set to 2 ($\varepsilon=2$). Dotted blue-line represents results for $\varepsilon=1$ (same as in (a)). (c) Enlarged subplot (b). Shows that the difference between $\varepsilon=1$ and $\varepsilon=2$ for the dielectric-spacer is marginal.

To show that the dependence on the dielectric-spacer permittivity $\varepsilon$ is marginal we plotted it for $\varepsilon=1$ and $\varepsilon=2$ for the same set of inverted-dipole equivalent-circuit parameters. Fig. S5 (a) shows results for $\varepsilon=1$, Fig. S5 (b,c) show comparison of $\varepsilon=1$ (dotted lines) with $\varepsilon=2$ (solid lines). The difference between the absorption diagrams is minimal indicating that taking the actual permittivity of the dielectric-spacer into account is not necessary for the absorption-diagram analysis.

## S6. Resonance Modes in the Top Metal Layer

To illustrate the behavior of the modes for each of the considered resonator geometries we extracted the plots of electric field, magnetic field and current density from the CST Studio.





Fig. S6 Instantaneous electric filed (left), magnetic field (center) and current (right) for the mode at resonance, calculated for structures presented in Fig. S3. For U-shape, interconnected U-shape and Inverted-cross distance to the backplane was chosen to be 300 nm, for the cross it was chosen to be 2100 nm. Polarization of the incident electric field is shown in the upper right corner.

Fig. S6 shows electric field, magnetic field and current in the top metal layer at resonance for four different resonator types. One can see that for the interconnected U-shape large portion of the current oscillates with the same pattern as for non-interconnected U-shape, however there is also part of the current that is floating throughout the structure similar to that for the inverted cross.





Fig. S7 Amplitude of electric filed for the four types of resonators in a vertical cross-section plane passing through a center of each structure. In the upper left corner of each subplot polarization of the incident electric field as well as the plot cross-section orientation are shown.

To illustrate the allocation of modes in the Z-direction we plot Fig. S7 that shows electric field amplitudes for the four resonator types in a vertical cross-section. Triangles on each colorbar mark the maximal field amplitude achieved in the structure (full 3D-model, not only the cross-section). Amplitude of the filed in the incident plane wave is ${10}^{7}$ V/m. The maximal field-amplitude enhancement factor for all structures is between 42 and 71.

## S7. Polarization Dependence of the CMOS-produced Structures

Although the experimental setup does not allow to distinguish between the linear light polarizations, it is possible to do it via the full-wave simulation model. Since the results of the experimental and simulated structure overlap well with one another for the unpolarized light, it is expected that the simulation results for polarized light will be reliable as well.





Fig. S8 Simulation results for the CMOS-produced structure for TE, TM and unpolarized light. The absorption is polarization-independent until approximately 50 degrees of incidence.

Fig. S8 shows polarization dependence of the absorption of the CMOS-produced structure. The absorption polarization independence holds up until around 50 degrees of incidence. After 50 degrees the absorption peak splits into two and the peak value decreases.
